# Efficient malic acid production from glycerol with *Ustilago trichophora* TZ1

**DOI:** 10.1186/s13068-016-0483-4

**Published:** 2016-03-17

**Authors:** Thiemo Zambanini, Eda Sarikaya, Wiebke Kleineberg, Joerg M. Buescher, Guido Meurer, Nick Wierckx, Lars M. Blank

**Affiliations:** Institute of Applied Microbiology-iAMB, Aachen Biology and Biotechnology-ABBt, RWTH Aachen University, Worringerweg 1, 52074 Aachen, Germany; BRAIN AG, 64673 Zwingenberg, Germany

**Keywords:** Adaptive laboratory evolution, Glycerol, Malate, Medium optimization, *Ustilago trichophora*

## Abstract

**Background:**

The large surplus of crude glycerol, as main low-value waste stream in biodiesel production, has led to the investigation of new possibilities for the production of value-added chemicals from this feedstock. New and efficient (bio-) catalysts are needed that are able to convert glycerol to versatile chemical building blocks. This would contribute to further develop away from a mainly petroleum based, to a sustainable, bio-based industry. One promising group of discussed building block chemicals are dicarbonic acids.

**Results:**

Here, we report the efficient synthesis of malate from glycerol using *Ustilago trichophora* RK089, which was identified in a screening of 74 Ustilaginaceae. For economically feasible production that can compete with existing processes, a high productivity is required. By adaptive laboratory evolution, the growth and production rate were increased by 2.5- and 6.6-fold, respectively. Further medium optimization increased the final titer, yield, and overall production rate to 196 g L^−1^, 0.82 g_mal_ g_gly_^−1^, and 0.39 g L^−1^ h^−1^, respectively.

**Conclusions:**

This titer is the highest reported for microbial malate production, making *U. trichophora* TZ1 a promising microbial production host for malate from crude glycerol, especially since it is not genetically engineered. Since this production process starts from an industrial waste stream as substrate and yields an interesting platform chemical, which can be used to replace petro-chemicals, it greatly contributes to a sustainable bio-economy.

## Background

In recent years, it has become apparent that a switch from our mainly petrochemical-based industry toward a bio-based, carbon neutral economy is inevitable. This switch requires new precursors for many different chemicals in a broad range of sectors, such as the mobility, polymer, food additives, and pharmaceutical industries, which still rely mainly on fossil resources.

One chemical of interest is the C4 dicarbonic acid malic acid, which has been used as acidulant in foods and beverages for decades [1]. Malate has great potential as building-block chemical, for instance as a bio-based precursor for maleic anhydride, or for substituted tetrahydrofuran derivatives [2, 3]. It can also be used for the production of bio-degradable polymers [4]. In 2004, Werpy and Petersen considered 1,4-diacids (malate, succinate, fumarate) one of the twelve most promising chemicals to produce from biomass [3]. Since it is, as intermediate of the tricarboxylic acid cycle, a natural product of many microbes, microbial production is considered to be promising [4].

Indeed, the possibility of microbial production of malate has already been known and investigated for a long time. In 1962, Abe et al. [5] selected *Aspergillus* *flavus* as production strain and patented the production process reaching a final titer of 58 g L^−1^ at a rate of 0.27 g L^−1^ h^−1^ and with a yield of 0.78 mol malate per mol glucose. However, *A.* *flavus* is known to produce aflatoxins excluding it as industrially applicable production strain, especially for food-grade malate [6]. To circumvent such problems, well-established model organisms, such as *Saccharomyces* *cerevisiae*, *Aspergillus* *niger*, and *Escherichia* *coli*, were engineered for microbial malate production [7–10]. These efforts resulted in production values comparable to the ones with *A.* *flavus*. In 2013, Brown et al. [11] reported a production process for malate with *Aspergillus* *oryzae* producing 154 g L^−1^ malate with a rate of 0.94 g L^−1^h^−1^ and a yield of 1.38 mol mol^−1^ on glucose. *A. oryzae* is a close relative to *A.* *flavus* which produces no aflatoxins and is generally regarded as Safe (GRAS).

However, production processes with *Aspergillus* species have certain drawbacks, such as the filamentous growth, which results in difficulties with the oxygen supply during large-scale fermentation [12]. Therefore, a new, unicellular production strain would be favorable. In 2014, Geiser et al. [13] screened 68 Ustilaginaceae for the production of organic acids. They found many strains from this family to produce malate naturally, besides other organic acids, such as succinate or itaconate. Ustilaginaceae are a family of plant pathogenic fungi, of which the haploid form grows unicellularly. Further, they show tolerance to high concentrations of organic acids and they do not produce toxins, which makes them industrially applicable, even for the food industry.

Thus far, most malate production studies have focused on glucose as a substrate. Recently, glycerol has been heralded as new substrate for the production of chemicals [14]. The rising production of biodiesel, 123 million tons per year predicted by 2016 [15], is accompanied by the production of around 19 million tons per year of crude glycerol as main waste stream (10 % (w/v)). Although this glycerol itself has been considered one of the most important building blocks to be produced from biomass [16], the large volume of crude glycerol side streams has become a burden rather than a blessing. The overall process of biodiesel production would become economically more favorable, if new applications for the resulting crude glycerol were found. One application discussed frequently over the last years is the microbial conversion of the crude glycerol to value-added chemicals [14, 17]. Different production processes using glycerol as precursor have been reported, such as the production of 1,3-propanediol [18, 19], polyhydroxyalkanoates [20], lipids [21], succinate [22], citrate [23], and erythritol [24]. The possibility of malate production from glycerol, however, has thus far only been proposed, but not investigated [17]. One advantage of the microbial conversion of glycerol to C4 dicarboxylic acids, such as malate or succinate is the possibility of CO_2_ fixation through the action of pyruvate carboxylase. By this reaction, the three-carbon pyruvate and CO_2_ are converted to the four-carbon oxaloacetate, theoretically enabling a process with a net carbon fixation [17].

In this study, we present the yeast-like growing smut fungus *Ustilago* *trichophora* TZ1 as new production host for malate from glycerol, combining high productivity with little by-product formation and avoidance of consumer opinion and regulatory restrictions, due to production with a genetically not modified organism.

## Results and discussion

### Selection and evolution of *Ustilago* *trichophora* as best producer of malate from glycerol

**Fig. 1:**
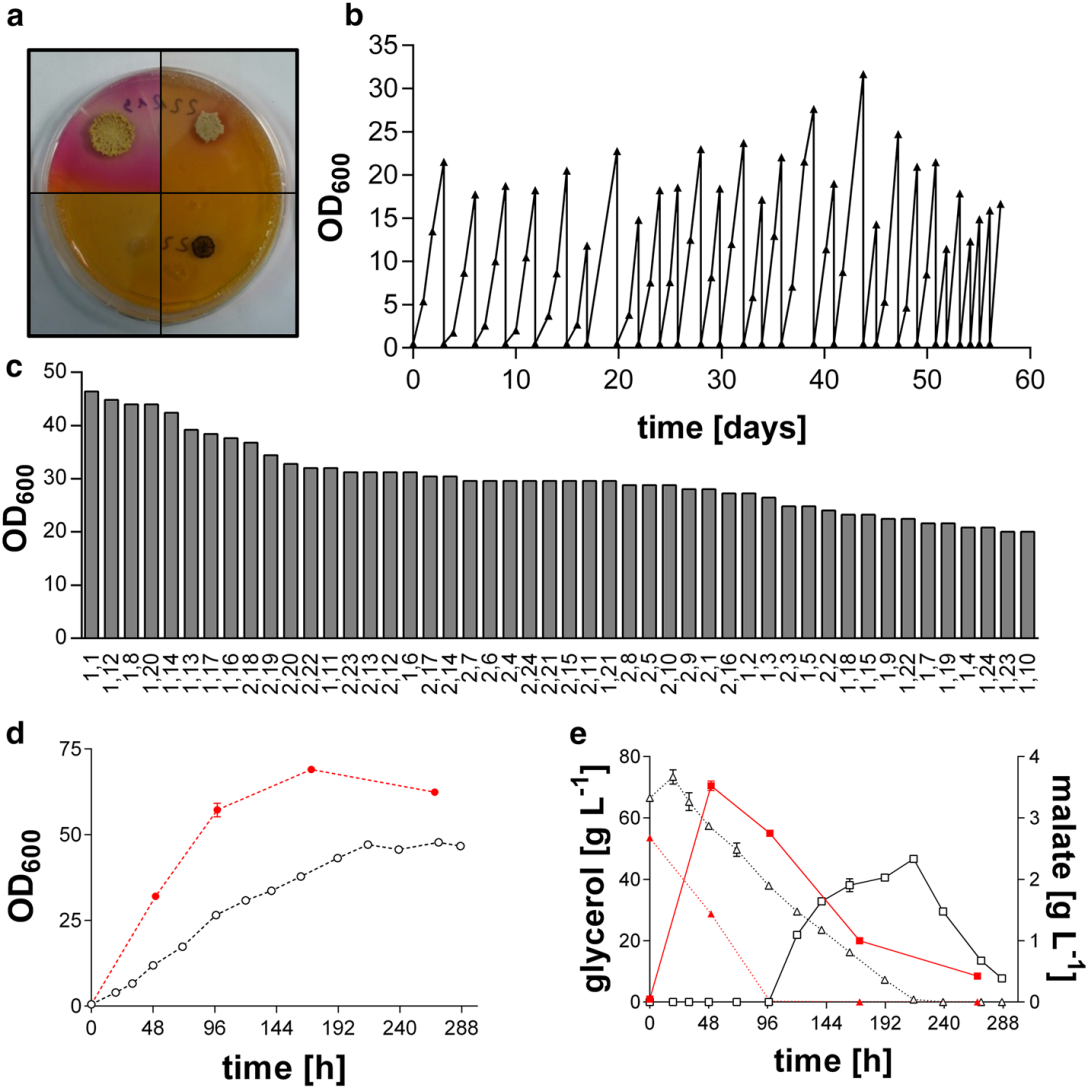
Screening and ALE for the production of organic acids from glycerol. **a** Four Ustilaginaceae in solid medium screening with glycerol as sole carbon source and methyl red as pH indicator, *pink halos* indicate acid production, **b** ALE of *U.* *trichophora* on glycerol as sole carbon source. A single representative culture is shown, **c** Rank ordered OD_600_ after 24 h of 48 single colonies from the two parallel *U.* *trichophora* TZ1 cultures, the first numbers on the *X-axis* indicate from which parallel line the clone is derived, **d** Comparison of growth for *U.* *trichophora* TZ1 (*closed symbols*, *red*) and wild type (*open symbols*, *black*) on glycerol, **e** Comparison of acid production (*squares*, *solid lines*) and glycerol consumption (*triangles*, *dotted lines*) for *U.* *trichophora* TZ1 (*closed symbols*, *red*) and wild type (*open symbols*, *black*)

Ustilaginaceae are known to produce a broad variety of secondary metabolites and other products from glucose naturally, such as itaconate, malate, and succinate [13]. In order to identify strains that produce acids from glycerol efficiently, 74 Ustilaginaceae were initially screened on solid glycerol medium with methyl red as pH indicator. The seven best strains were chosen for further characterization based on growth rate (colony size) and acid production (pink halo) (Fig. 1a). Subsequently, these seven strains were assessed in more detail in liquid cultures in 2-(N-morpholino)ethanesulfonic acid (MES)-buffered modified Tabuchi medium (MTM) containing 50 g L^−1^ glycerol and 0.8 g L^−1^ NH_4_Cl. *U.* *trichophora* (CBS 131473) was selected as the best growing strain with a growth rate of 0.11 ± 0.00 h^−1^, producing 2.3 ± 0.1 g L^−1^ malate in 216 h at an overall rate of 0.01 ± 0.00 g L^−1^ h^−1^ (Fig. 1e). Although *U.* *trichophora* was the best growing and producing strain on glycerol out of the 74 screened strains, these values are low compared to growth and malate production of this strain on glucose under the same conditions (0.45 ± 0.02 h^−1^ and 0.08 ± 0.00 g L^−1^ h^−1^, respectively), indicating that its metabolic capacity has room for improvement on glycerol.

Adaptive laboratory evolution (ALE) is a method frequently used to improve different characteristics of microbes by adapting them to environmental conditions, such as sub-optimal pH-values or temperatures, different stress factors or the ability to utilize non-preferred carbon sources [25, 26]. Especially, the potential to improve the growth rate on non-preferred carbon sources has been shown in many studies. For instance, Sonderegger et al. [27] and Kuyper et al. [28] could improve the growth rate of engineered *S.* *cerevisiae* strains on xylose and Ibarra et al. [29] were able to double the growth rate of *E.* *coli* on glycerol at 30 °C by ALE. Using a simple re-inoculation scheme with two parallel shake flask cultures, as described in “Methods” section, the growth rate of *U.* *trichophora* was improved after 58 days with 27 re-inoculations, corresponding to approximately 140 generations (Fig. 1b). Screening 48 single colonies of the evolved cultures (Fig. 1c) resulted in a clonal culture with increased growth rate of 0.26 ± 0.03 h^−1^ for the best colony, *Ustilago* *trichophora* TZ1, which constitutes a 2.4-fold improvement over the wild type. It should be noted that, as known for Ustilaginaceae [13], the OD_600_ of *U.* *trichophora* generally continues to increase after nitrogen limitation (Fig. 1d), likely as a result of intracellular lipid accumulation and associated morphological changes [30]. The indicated growth rates were therefore assessed separately in cultures with a higher sample resolution in the initial growth phase (data not shown). The malate production reached 3.5 ± 0.1 g L^−1^ within 50 h, corresponding to a malate production rate of 0.07 ± 0.00 g L^−1^h^−1^ (Fig. 1e).

### Medium optimization increases malate production with *U. trichophora* TZ1

To further improve the malate production of *U.* *trichophora* TZ1, the influence of different medium components (MES and CaCO_3_ buffer; 10, 50, 100 mg L^−1^ FeSO_4_; 0.125, 0.25, 0.5, 1 g L^−1^ KH_2_PO_4_) was investigated in 24-deep well plates. Different FeSO_4_ concentrations were tested, since FeSO_4_ is known to influence organic acid production in *A.* *terreus* [31]. Influences of MES and CaCO_3_ buffer on organic acid formation in Ustilaginaceae were already shown by Geiser et al. [13] and the impact of KH_2_PO_4_ concentration on organic acid production has been shown by Jeon et al. [32]. Changing the FeSO_4_ and KH_2_PO_4_ concentrations did not influence growth or malate production for *U.* *trichophora* TZ1 (data not shown). A change from MES buffer to CaCO_3_ buffer (33 g L^−1^), however, resulted in a higher titer of 5.3 ± 0.3 g L^−1^ malate after 98 h of cultivation upon glycerol depletion. In MES-buffered cultures, pH-values decreased during cultivation, while pH-values in cultures with CaCO_3_ stayed constant. Given the higher buffer capacity of CaCO_3_, it is likely that in MES-buffered cultures pH decreases to below the minimum for malate production of *U.* *trichophora* TZ1. A similar phenomenon was also observed for certain itaconate producing *Ustilago* strains [13]. Another advantage of CaCO_3_ as buffer could be the additional supply of CO_2_, since the microbial production of malate via pyruvate likely relies on CO_2_ as co-substrate. Upon reaction of malic acid with CaCO_3_, HCO_3_^−^ is formed, which is in equilibrium with dissolved CO_2_ dependent on the pH. This can provide an additional HCO_3_^−^ supply for pyruvate carboxylase to form oxaloacetate from pyruvate. Indeed, feeding of additional CO_2_ to an engineered malate producing *S. cerevisiae* strain significantly improved malate production [33]. By this, the malate production can theoretically be enhanced to yield 1 mol malate per mole of glycerol [7].

**Fig. 2:**
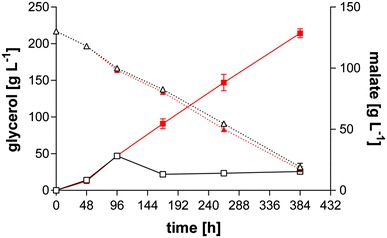
Shake flask cultivation of *U.* *trichophora* TZ1 in MTM with 200 g L^−1^ glycerol. Cultures contained 100 g L^−1^ CaCO_3_. Comparison of acid production (*squares*, *solid lines*) and glycerol consumption (*triangles*, *dotted lines*) for samples dissolved with HCl (*closed symbols*, *red*) and supernatant (*open symbols*, *black*). *Error bars* indicate deviation from the mean (*n* = 2)

Since the malate production rate did not decrease until glycerol depletion, the initial glycerol concentration was increased to 200 g L^−1^. In these cultures, the malate concentration reached 129 ± 5 g L^−1^ upon glycerol depletion (Fig. 2). This concentration was only observed upon dissolution of solid medium components with HCl prior to filtering for HPLC analysis. If HCl addition was omitted, the concentration reached 28 ± 2 g L^−1^ after 96 h of cultivation, after which it dropped to 14 ± 1 g L^−1^ and stayed constant throughout cultivation. These results clearly show that the produced malate in combination with CaCO_3_ forms Ca-malate, which precipitates after a brief super-saturation to its solubility limit. This solubility is somewhat higher than reported values (approximately 10 g L^−1^) [34], which is likely due to differences in temperature and the presence of cells and other buffering agents.

### Higher initial glycerol concentration further increases malate production

**Fig. 3:**
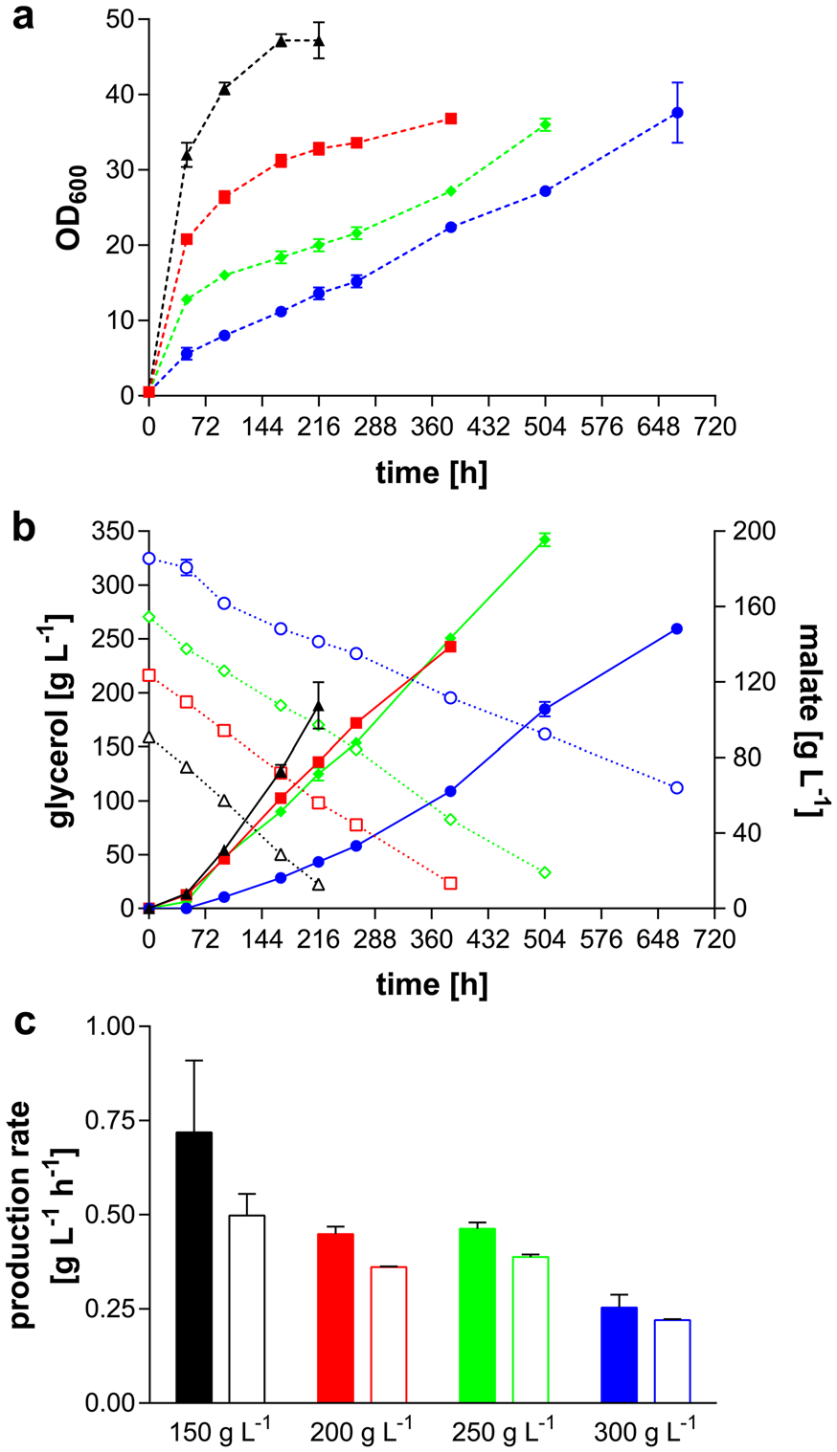
Shake flask cultivation of *U.* *trichophora* TZ1 in MTM with differing glycerol concentrations. Cultures contained 100 g L^−1^ CaCO_3_. **a** Comparison of growth. **b** Comparison of acid production (*solid lines*, *closed symbols*) and glycerol consumption (*dotted lines*, *open symbols*). 150 g L^−1^ (*triangles*, *black*), 200 g L^−1^ (*squares*, *red*), 250 g L^−1^ (*diamonds*, *green*), 300 g L^−1^ (*circles*, *blue*). **c** Maximal (*filled bars*) and overall (*open bars*) production rate per glycerol concentration. *Error bars* indicate deviation from the mean (*n* = 2)

To further investigate the influence of starting glycerol concentrations on malate formation, the initial glycerol concentration was varied between 150 and 400 g L^−1^ in 50 g L^−1^ increments (Fig. 3). Growth decreased with increasing initial glycerol concentrations (Fig. 3a), leading to complete growth inhibition at concentrations exceeding 300 g L^−1^ (data not shown). An initial glycerol concentration of 150 g L^−1^ led to the highest overall volumetric production rate of 0.50 ± 0.08 g L^−1^ h^−1^. Furthermore, malate production rates and glycerol uptake rates remained constant until depletion (Fig. 3b). Due to handling issues (i.e., shaking of viscous liquid), samples for 300 g L^−1^ could not be taken after 672 h. Hence, the maximal malic acid titer of 196 ± 5 g L^−1^ was reached with 250 g L^−1^ glycerol as starting concentration after 504 h, corresponding to an overall production rate of 0.39 ± 0.01 g L^−1^ h^−1^ (Fig. 3b). This culture also had the highest yield of 0.82 ± 0.02 g_mal_ g_gly_^−1^ (= 0.57 ± 0.01 mol_mal_ mol_gly_^−1^) although in general the cultures did not show large differences in yield and no clear trend could be observed (average for all cultures: 0.74 ± 0.9 g_mal_ g_gly_^−1^, which equals 0.51 ± 0.06 mol_mal_ mol_gly_^−1^). Interestingly, the rate in the culture with 250 g L^−1^ glycerol as starting concentration is only 22 % lower than the rate achieved with 150 g L^−1^ glycerol, even though the overall OD_600_ of the former culture is less than half of the latter. It seems that with high glycerol concentrations, the specific production rate per gram biomass is higher, although quantification is difficult due to possible differences in intracellular lipid formation, which significantly influences the biomass composition of *Ustilago* under these conditions [30, 35].

### Separation of growth and production

**Fig. 4:**
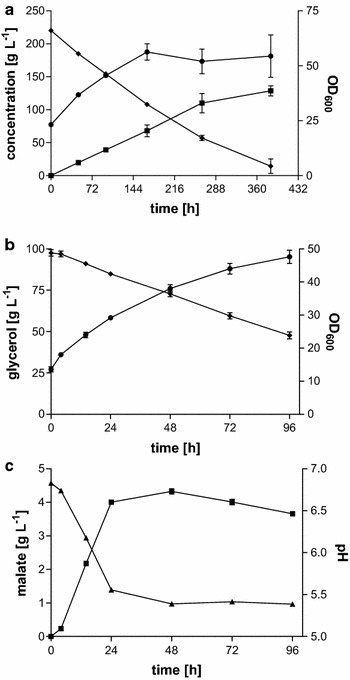
Malic acid production of *U.* *trichophora* TZ1 cells in shake flasks with aqueous glycerol solutions. Cultures contained either 200 g L^−1^ glycerol buffered with 100 g L^−1^ CaCO_3_ (**a**), or 100 g L^−1^ glycerol buffered with 100 mM MES buffer (**b**, **c**). OD_600_ (*circles*), malic acid concentration (*squares*), glycerol concentration (*diamonds*), and pH-value (*triangles*). *Error bars* indicate deviation from the mean (*n* = 2)

Ustilaginaceae and other fungi generally only initiate organic acid production upon depletion of an essential nutrient [35, 36], which poses an inherent trade-off between biomass and product formation. In order to investigate this trade-off, as well as to establish the minimal set of compounds needed during the malic acid production phase, cells grown for 24 h in 50 mL MTM containing 0.8 g L^−1^ NH_4_Cl and 50 g L^−1^ glycerol were centrifuged, washed twice with demineralized water, and transferred to 50 mL of an aqueous solution of 100 or 200 g L^−1^ glycerol. The resting cell conversion was either buffered with 100 g L^−1^ CaCO_3_ (200 g L^−1^ glycerol), 100 mM MES (Fig. 4), or not buffered at all (100 g L^−1^ glycerol) (data not shown).

In the CaCO_3_-buffered conversion, a concentration of 129 ± 11 g L^−1^ malic acid was reached after 384 h corresponding to a production rate of 0.34 ± 0.03 g L^−1^ h^−1^ (Fig. 4a), which equals the production rate of normal cultivations with 200 g L^−1^ initial glycerol (compare Fig. 2). In the MES-buffered system, the resulting titer was significantly lower, reaching 4.01 ± 0.08 g L^−1^ (Fig. 4c), while the unbuffered control produced only 20 ± 1 mg L^−1^. Both in the MES-buffered and in the unbuffered system, the pH dropped below 5.5 and 4.5, respectively, in contrast to the CaCO_3_-buffered system, which stayed above pH 6.0 throughout the production. These data indicate that during the production phase, no supplements in the medium are needed. The pH, however, is a critical parameter. In 2014, Geiser et al. [13] were already able to show the significant influence of buffer capacity on acid production with Ustilaginaceae. They cultivated Ustilaginaceae in differing concentrations of MES–buffer. Comparable to our data, a drop in pH inhibited further acid production. Apparently, a pH greater than 5.4 is needed for efficient malate production with *U.* *trichophora* TZ1. Interestingly, even without further malate production in the MES-buffered system the glycerol consumption rate stays constant for 72 h. This suggests the activity of an overflow metabolism, possibly switching to alternative products, such as extracellular glycolipids, polyols, or CO_2_. Additionally, with both buffers, OD_600_ increases more than 2.5-fold, even though no source of nitrogen, phosphate, or trace elements was present. This increase in OD_600_ can for a large part be attributed to the production of intracellular lipids [30]. A significant increase in dry cell weight after nitrogen limitation has also already been reported by Klement et al. [35] in 2012. They could show that the cell number still increased by about 30 % after limitation. However, the carbon-to-nitrogen weight ratio in the biomass increased from 5.9 in cells during unlimited growth to about 20 in cells during stationary phase, clearly showing that the increasing OD_600_ is a result of nitrogen “dilution.” Consequently, one additional division cycle is possible after nitrogen depletion, which probably does not positively affect the bio-catalytic potential, since the total amount of proteins remains the same [35]. In addition, a significant amount of glycerol is used for lipid formation, which mainly occurs after nitrogen depletion. Although this generally detracts from the overall efficiency of malate production, the lipids themselves are a useful secondary product for food, cosmetic, or biofuel applications, and their inclusion in the cells makes them relatively easy to separate. These phenomena may partly explain the yield of 0.43 ± 0.00 mol_mal_ mol_gly_^−1^, which is lower than the yield in cultures with complete mineral media containing 200 g L^−1^ glycerol (0.49 ± 0.00) even though no glycerol is needed for biomass production. In addition, the lack of micronutrients likely serves as an additional stress factor which reduces the cells’ productivity and tolerance to malate.

In all, these results indicate the high potential of *U.* *trichophora* TZ1 as production organism for malic acid. Although the overall production rate of 0.50 ± 0.08 g L^−1^ h^−1^ is lower than reported rates for other organisms [11], a titer of almost 200 g L^−1^ is reached with a strain that is not genetically modified. To our knowledge, this titer is the highest reported value for microbial malic acid production. In the future, this process can be taken to bioreactors for further improvement, making full use of *U.* *trichophora*’s unicellular growth, focusing on increasing the production rate and final titer while circumventing handling and downstream processing issues associated with CaCO_3_ cultures. These issues include problems of oxygenation by shaking due to high viscosity and the general drawback of a huge gypsum waste stream for industrial scale processes resulting from production processes involving CaCO_3_ as buffering or downstream processing agent.

By this, the overall production process for malic acid with *U.* *trichophora* could be further improved, making *U.* *trichophora* a promising industrially applicable production organism for malic acid.

## Conclusions

The microbial conversion of glycerol to value-added chemicals has been the focus of research for many years. The identification and optimization of *U.* *trichophora* TZ1 as efficient malate producer opens up novel opportunities for glycerol valorization, potentially adding to the overall feasibility of a biodiesel bio-refinery. The reached titer of almost 200 g L^−1^ is the highest titer reported for any microbial malic acid production, and further improvements in the production rate and yield can be expected from process optimization and metabolic engineering. Especially, the generation of a closed carbon-balance would shed light on possible targets, since it would clarify the amount of glycerol used for by-product formation and respiration. Abovementioned facts reveal the potential for further research and improvement of *U.* *trichophora* TZ1 as promising, industrially applicable production organism for malic acid, or as a gene donor of interest for heterologous malate producers. This confirms in general the potential of the Ustilaginaceae for bio-catalysis.

## Methods

### Strains and culture conditions

The 68 strains belonging to the family Ustilaginaceae screened by Geiser et al. [13] in 2014, except for *Ustilago* *avenae* CBS 131466 (2216), plus *Ustilago* *maydis* DSM 3121 (1949), *U.* *maydis* DSM 4500 (1950), *U.* *maydis* DSM 14603 (1951), *U.* *maydis* Nr. 483 ATCC 22902 (21702), *U.* *maydis* Nr. 495 ATCC 22914 (2179), *U.* *trichophora* CBS 131473 (2219), and *Ustilago* *hordei* Uh4875-4 Mat1 [37] were screened in this study. The numbers in parenthesis indicate in-house strain numbers.

As standard medium, MTM was used according to Geiser et al. [13] with 0.2 g L^−1^ MgSO_4_ 7 H_2_O, 10 mg L^−1^ FeSO_4_ 7 H_2_O, 0.5 g L^−1^ KH_2_PO_4_, 1 mL L^−1^ vitamin solution, 1 mL L^−1^ trace element solution, and 0.8 g L^−1^ NH_4_Cl and 50 g L^−1^ glycerol, unless stated otherwise. As buffer, either 100 mM MES or differing concentrations of CaCO_3_ were used. When using solid CaCO_3_ buffer, the concentration of medium components is always based on the total volume of liquid and solid.

For solid medium screening, plates with MTM containing 20 mM MES pH 6.5, 2 % (w/v) Agar–Agar, and 0.02 g L^−1^ methyl red were used. 10 µL of an overnight culture, grown in MTM with 10 g L^−1^ glucose and 100 mM MES was spotted on the plates in duplicates and the plates were incubated at 30 °C for 9 days.

For adaptive laboratory evolution, *U. trichophora* was grown in MTM with 100 mM MES in 100-mL Erlenmeyer flasks with 10 % (v/v) filling volume. OD_600_ was measured daily until an OD_600_ of >16 was reached, after which a new culture was inoculated to an OD_600_ of 0.5. This procedure was repeated sequentially for 57 days. Growth rates of evolved and original strains were assessed in separate cultures in MTM with CaCO_3_ as buffer.

Medium optimization was performed in 24-deep well plates (Enzyscreen, System Duetz^®^) with 1.5 mL MTM containing either MES or CaCO_3_ and differing concentrations of FeSO_4_ and KH_2_PO_4_ incubated at 30 °C (relative air humidity = 80 %) shaking at 300 rpm (shaking diameter = 50 mm).

Shake flask production experiments were conducted in 500-mL Erlenmeyer flasks with 10 % (v/v) filling volume. All cultures were incubated at 30 °C (relative air humidity = 80 %) shaking at 200 rpm (shaking diameter = 25 mm). As preculture, MTM with 100 mM MES was inoculated from an overnight YEP culture and grown over night. All shake flask cultures were inoculated to a starting OD_600_ of 0.5. All yields were calculated based on the actual amount of glycerol consumed.

### Analytical methods

All experiments were performed in duplicates. Shown is the arithmetic mean of the duplicates. Error bars and ± values indicate deviation from the mean.

When using CaCO_3_ as buffer, 1 mL of culture broth was taken for OD_600_ determination and HPLC analysis. The CaCO_3_ was dissolved with HCl prior to further measurements. OD_600_ was determined in an Ultrospec 10 cell density meter (Amersham Biosciences, UK), samples were diluted to an OD_600_ between 0.1 and 0.8.

For HPLC analysis, centrifuged samples (13.000 g, 5 min) were filtered through cellulose acetate filters (diameter 0.2 µm, VWR, Germany) and subsequently diluted 1:10 with distilled water. Glycerol and organic acids were analyzed on a Dionex Ultimate 3000 HPLC (Dionex, USA) with an Organic Acid Resin column (CS–Chromatographie, Germany) kept at 75 °C, with a constant flow rate of 0.8 mL min^−1^ of 5 mM sulfuric acid as eluent. For detection, a Shodex RI 101 detector at 35 °C and a variable wavelength UV detector (Dionex, USA) at 210 nm were used.

Ammonium concentration was determined by a colorimetric assay according to Willis [38].

## Abbreviations

MTM: modified Tabuchi medium
ALE: adaptive laboratory evolution
MES: 2-(N-morpholino) ethanesulfonic acid
HPLC: high-performance liquid chromatography
